# Gαi protein subunit: A step toward understanding its non-canonical mechanisms

**DOI:** 10.3389/fcell.2022.941870

**Published:** 2022-08-24

**Authors:** Soraya Villaseca, Gabriel Romero, María J. Ruiz, Carlos Pérez, Juan I. Leal, Lina M. Tovar, Marcela Torrejón

**Affiliations:** Laboratory of Signaling and Development, Group for the Study of Developmental Processes (GDeP), Department of Biochemistry and Molecular Biology, Faculty of Biological Sciences, University of Concepción, Concepción, Chile

**Keywords:** heterotrimeric G-protein, signaling, migration, asymmetric cell division, cell polarity

## Abstract

The heterotrimeric G protein family plays essential roles during a varied array of cellular events; thus, its deregulation can seriously alter signaling events and the overall state of the cell. Heterotrimeric G-proteins have three subunits (α, β, γ) and are subdivided into four families, Gαi, Gα12/13, Gαq, and Gαs. These proteins cycle between an inactive Gα-GDP state and active Gα-GTP state, triggered canonically by the G-protein coupled receptor (GPCR) and by other accessory proteins receptors independent also known as AGS (Activators of G-protein Signaling). In this review, we summarize research data specific for the Gαi family. This family has the largest number of individual members, including Gαi1, Gαi2, Gαi3, Gαo, Gαt, Gαg, and Gαz, and constitutes the majority of G proteins *α* subunits expressed in a tissue or cell. Gαi was initially described by its inhibitory function on adenylyl cyclase activity, decreasing cAMP levels. Interestingly, today Gi family G-protein have been reported to be importantly involved in the immune system function. Here, we discuss the impact of Gαi on non-canonical effector proteins, such as c-Src, ERK1/2, phospholipase-C (PLC), and proteins from the Rho GTPase family members, all of them essential signaling pathways regulating a wide range of physiological processes.

## Introduction

Heterotrimeric G-proteins are the biggest signaling cores, acting as molecular switches that control the movement of information resulting from a variety of extracellular cues to the several intracellular effectors that control cell behavior (Gilman, 1987; Morris and Malbon, 1999). The heterotrimeric G-proteins are constituted by three subunits (Gα, Gβ, and Gγ) that cycle between a Gα GDP-bound form/Gβγ (OFF state) and a Gα GTP-bound form dissociated from the Gβγ dimer (ON state) (Birnbaumer, 2007). This change between the OFF and ON states is due to a conformational change in the intracellular portion of the protein after the binding of a specific ligand in the extracellular domain of the GPCR (G protein coupled receptor) (Goldsmith and Dhanasekaran, 2007). The active species (Gα-GTP and Gβγ dimer) can also interact with different effector proteins, regulating different signaling pathways (Sato et al., 2006). In addition, the activity of Gα subunits are regulated mainly through three classes of proteins: 1) GTPase activating proteins (GAPs) are negative regulators of G protein signaling, 2) guanine nucleotide-dissociation inhibitors (GDIs) inhibit dissociation of GDP from Gα subunits, and 3) guanine nucleotide-exchange factors (GEFs) that can induce guanine nucleotide exchange (GDP for GTP), activating the Gα subunit itself (Jaffe and Hall, 2005; Guilluy et al., 2011). G protein families are classified by their Gα subunits and are grouped into four families by their sequence and functional similarities: Gαs/olf, Gαi/o/z, Gαq/11, and Gα12/13 (Wilkie et al., 1992; Krishnan et al., 2015; Downes and Gautam, 1999).

**FIGURE 1:**
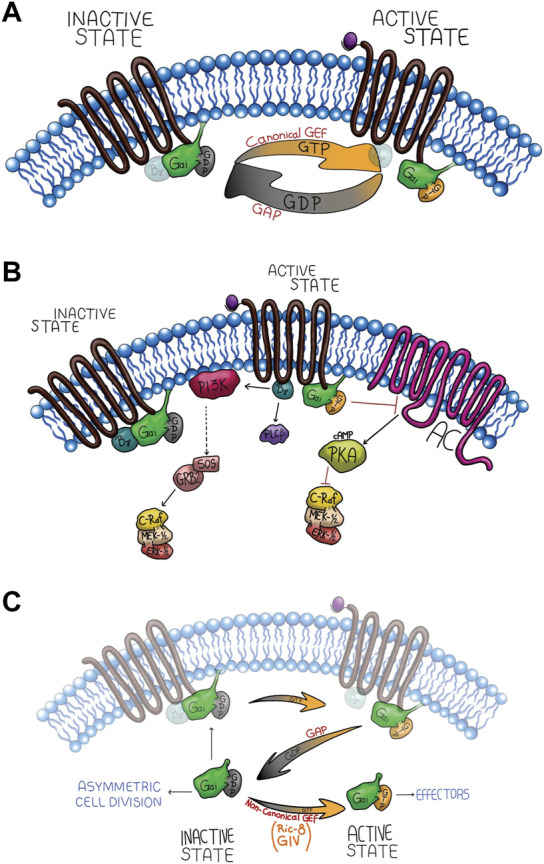
Gαi canonical signaling network through receptor activation **(A,B)** and non-canonical activation **(C)**. **(A)** The upper panel shows the G-protein canonical signaling activation by the GPCR GEF activity, generating two active species (Gαi-GTP and Gβγ released). The intrinsic GTPase activity of the Gαi subunit hydrolyze GTP to GDP, promoting its re-association with Gβγ. **(B)** Canonical signaling pathway of Gαi, which is inhibitory of Adenylyl Cyclase (AC), and Gβγ activates MAPK signaling cascade. **(C)** The non-canonical activation signaling. During this process, Gαi interacts with cytosolic GEFs such as Ric-8 or GIV which stimulate the GDP/GTP exchange, activating Gαi in a receptor-independent manner potentiating the canonical signal or activating non-canonical effectors.

Gαi is the largest and most diverse family of Gα subunits and includes Gαi1, Gαi2, Gαi3, Gαo, Gαt, Gαg, and Gαz, all sensitive to pertussis toxin (PTX) (Malbon, 2005). Three Gαi isoforms have been described in mammals, including Gαi1, Gαi2, Gαi3, best known as “the inhibitory Gα subunits,” suppressing adenylyl cyclase activity, resulting in decreased intracellular cycle-AMP (cAMP) levels (Figures 1A,B) [Wong et al., 1991; Xiao et al., 1999; Pines et al., 1986]. As we shall see in this Review, despite that Gαi subunits are generically classified by their ability to “inhibit adenylyl cyclase,” these three isoforms have other cAMP-independent functions. Gαi proteins were first described localized exclusively at the plasma membrane, although cell fractionation and immunofluorescence studies, were key tools demonstrating that a fraction of Gαi proteins were intracellularly identified and even free of Gβγ, suggesting the existence of non-canonical signaling pathways for Gαi subunits (Stow et al., 1991; Schurmann et al., 1992; Pimplikar and Simons 1993; Wilson et al., 1993; Muller et al., 1994; Montmayeur and Borrelli 1994; Ogier-Denis et al., 1995; Maier et al., 1995; Denker et al., 1996; Lin et al., 1998; Marrari et al., 2007). Supporting this intracellular location for Gαi subunits, we found studies by fluorescence and EPR (Electron Paramagnetic Resonance), showing that the myristoylated amino terminus, a key lipid post-translational modification, sign for this Gαi family, presents and intramolecular interaction with its surface in the GαGTP-bound state (Preininger et al., 2003). Gαi membrane location is mediated by two lipid modification, myristoylation and palmitoylation at the amino terminus. Gαi family is the only family that is myristoylated, in contrast with the other Gα (Gαs, Gα12/13, and Gαq) families that are only palmitoylated, both lipid modifications are important to allow interaction with the membrane, in order to interact with their receptor and with their membrane effectors (Chen and Manning, 2001; Morales et al., 1998; Fishburn et al., 1999). Myristoylation has been also found important for palmitoylation of Gαi (Dunphy et al., 1996). Thus, for Gαi non-canonical functions that required an intracellular location, Gαi may rearrange its structure hiding its lipid modification increasing its solubility, thus allowing interaction with other non-membrane effectors (Preininger et al., 2003).

Within the non-canonical function for Gαi proteins, we found a critical role on Golgi structures and function (Jamora et al., 1999; Yamaguchi et al., 2000), as well as controlling steroids receptors and tyrosine kinase receptor signaling pathway (Kreuzer et al., 2004; Kumar et al., 2007), and regulating the mitotic spindle positioning during asymmetric cell division by interacting with GPR/GoLoco proteins (Yu et al., 2000; Gotta and Ahringer 2001; Parmentier et al., 2000; Schaefer et al., 2001), the latest suggesting an important role during early development. Furthermore, studies using PTX to inhibit Gαi mediated signaling and recently by gene targeted mice have shown that Gαi has a non-redundant and a critical role in leukocyte migration. Thus, the active form of Gαi1 can regulate the migration and cellular adhesion of immune cells, such as neutrophils (Surve et al., 2016; To and Smrcka, 2018), and Gαi2 can control macrophage and T lymphocyte migration in a GPCR-dependent and independent manner (Wiege et al., 2012; Hwang et al., 2007). The depletion of Gαi2 contributes to inflammatory bowel disease, and Gαi3 is needed to block the insulin antiautophagic action in mouse liver whereas deletion of both Gαi2 and Gαi3 in mice leads to death *in utero* (Gohla et al., 2007).

Although, the canonical activation of Gα subunits through ligand-GPCR complexes (canonical GEF activity) (Figure 1A) is well-established (Pierce et al., 2002), Gα subunit activity could also be regulated by several receptor-independent G protein activators (AGS: activators of G-protein signaling) classified into three groups: group I activates the Gαi/o subunit as a guanine exchange factor (Figure 1C), Group II has GPR motifs that stabilize the GDP-bound conformation of the Gα subunit (Cismowski et al., 2001), and group III bind Gβγ to dissociate it from the Gα subunit (Blumer et al., 2006a). Group I and II has been implicated in Gαi AMPc-independent signaling pathways controlling mitotic spindle dynamics during asymmetric cell division, polarity, growth, differentiation, and pathological processes, such as cancer (Feigin and Muthuswamy, 2009). Understanding how Gαi controls cell polarity is essential to develop new strategies to impair cancer progression, treat developmental defects, and tissue regeneration. Therefore, in this review, we discuss and summarize the implications of non-canonical pathways of Gαi proteins during cell polarity and several biological processes, such as proliferation, survival, tissue differentiation and cell migration.

## Gαi regulates asymmetric cell division

Several genetic studies in *Caenorhabditis elegans* embryos and *Drosophila neuroblasts* have reported that Gαi and Gαo subunits regulate apicobasal cell polarity in a receptor-independent manner (Bellaiche and Gotta, 2005; Siderovski and Willard, 2005).

**FIGURE 2:**
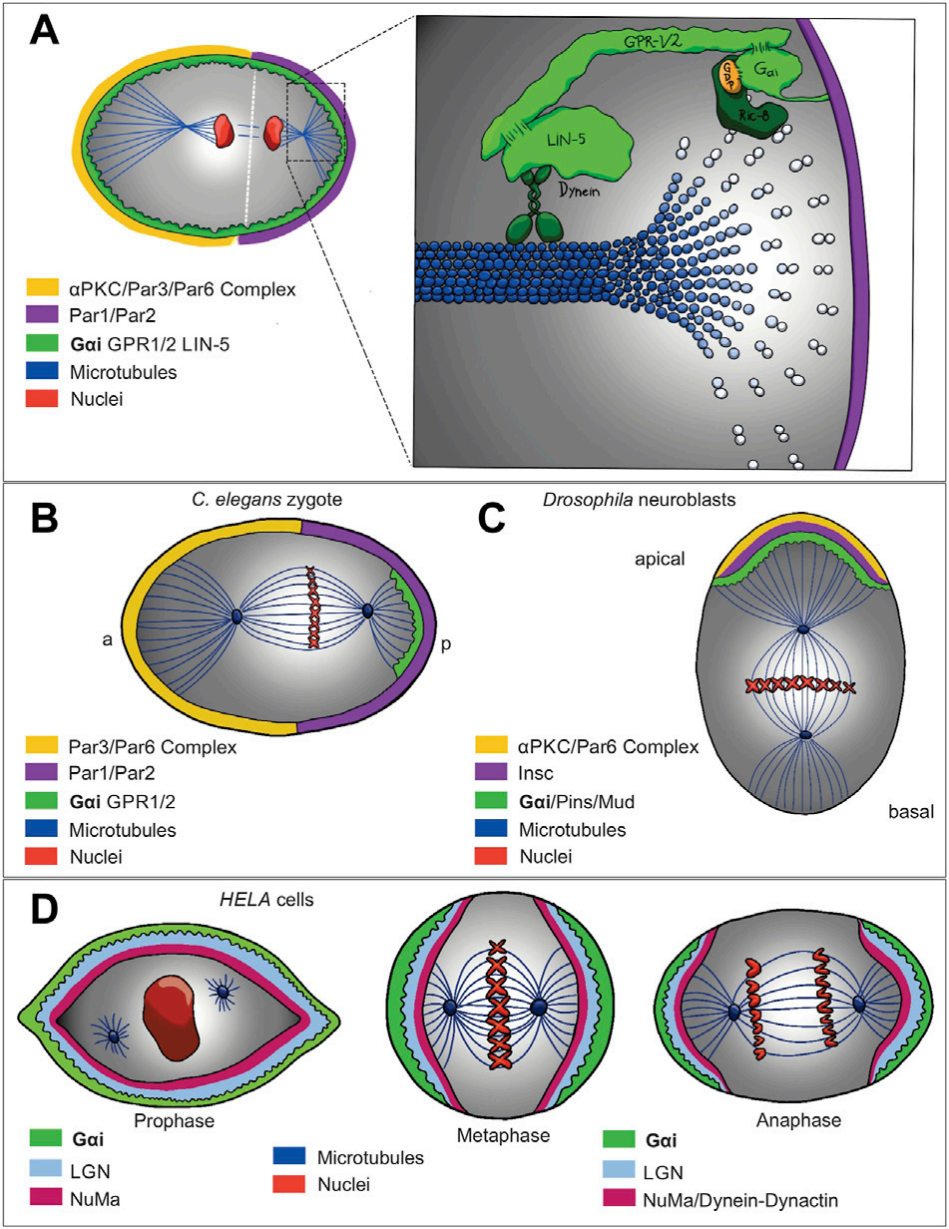
Gαi non-canonical signaling network models that show regulation of asymmetric cell division **(A)** in **(C)** elegans **(B)**, Drosophila neuroblast **(C)** and HELA cells **(D)**. Evolutionary conservation of a molecular complex composed of the Gαi subunit of the heterotrimeric G protein, LGN, dynein/dynactin complex and NuMA (respectively Gαi, Pins, and Mud in *Drosophila*, and GOA-1/GPA-16, GPR-1/2, and LIN5 in *C. elegans*) is localized at subcortical domain recruiting dynein, a motor protein, which also determines the movement along astral microtubules and generates pulling forces to orientate the spindle correctly. While Ric-8, a guanine nucleotide exchange factor, stimulates the exchange of GDP for GTP on the Gαi, triggering the dissociation of the complex that later, RGS activity stimulate the hydrolysis of GTP on Gαi, resulting in the Gαi-GDP reforming the Gαi-GDP/GPR-1/2 complex. On the other hand, Ric-8 as a scaffold protein, is required to localize Gαi and GPR-1/2 at the plasma membrane.

A variety of biological processes, such as asymmetric cell division and tissue morphogenesis require an apicobasal polarity, thus its alteration contributes to multiple diseases, including cancer (Feigin and Muthuswamy, 2009; Hirose et al., 2006; Knoblich, 2010). During asymmetric cell division, a non-canonical signaling pathway has been described for the Gαi subunit in which it regulates microtubule function during mitotic spindle positioning (Figure 2A). Asymmetric cell division has been studied in *C. elegans* one-cell embryo (Figure 2B), *Drosophila* embryonic neuroblasts (Figure 2C), and *Drosophila* sensory organ precursors, where the correct placement and asymmetry of the spindle give rise to daughter cells of different sizes and cellular function (di Pietro et al., 2016). Specifically, in *C. elegans*, asymmetric cell division is controlled by a protein complex that associate with the astral microtubules generating an imbalance in cortical forces thus asymmetrically positioning the mitotic spindle (Grill et al., 2001). Indeed, evolutionary conservation of a molecular complex composed of Gαi subunit from heterotrimeric G protein, leucine-glycine-asparagine (LGN), dynein/dynactin complex and nuclear and mitotic apparatus (NuMA) (respectively Gαi, partner of inscuteable (Pins), and mushroom body defect (Mud) in *Drosophila*, and guanine nucleotide-binding protein G (o) subunit (GOA-1)/G protein *α* subunit (GPA-16), G protein regulator 1/2 (GPR-1/2), and spindle apparatus lin-5 (LIN5) in *C. elegans*) is localized in a specific subcortical domain leading the recruitment of dynein (motor protein), which also determines the movement along astral microtubules and generates pulling forces to orientate the spindle correctly (Figure 2A) (di Pietro et al., 2016; Kiyomitsu, 2019; Poon et al., 2019). The Gαi subunit plays a crucial role during this process since inactivation of GOA-1 and GPA-16, as well as GPR-1/2 and Lin-5 result in a strongly reduced and symmetric pulling force (Figures 2A,B) (Kiyomitsu, 2019; Poon et al., 2019; Colombo et al., 2003; Gotta et al., 2003; Srinivasan et al., 2003).

In *Drosophila melanogaster* neuroblasts (NB), after delamination from the epithelium, the apically localized partitioning-defective 3 (Par3)/atypical protein kinase C (αPKC)/Par-6 complex recruits Inscuteable (Insc) to drive apicobasal polarity at the first asymmetric cell division (di Pietro et al., 2016). Gαi and Pins (the homologue of GPR-1/2), both recruited by Insc are also required for apical basal orientation of the mitotic spindle; therefore, Insc is a critical protein for asymmetric localization of Gαi and its biding partner, Pins (Figure 2C) (Schaefer et al., 2001). In contrast, mammalian Gαi as well as LGN and NuMA are crucial for the interaction between astral microtubules and cell cortex (Figure 2D) (Du and Macara, 2004; Woodard et al., 2010). Interestingly, in addition to Gαi/Pins, Gβγ is also involved in the control of spindle asymmetry in *Drosophila* NB (Fuse et al., 2003; Yu et al., 2003), and the overexpression of Gαi or Gβγ in NB leads to large or small symmetrical spindle formation, respectively (Fuse et al., 2003). However, considering that Pins acts as GDI for Gαi, by dissociating Gαi from Gβγ, the mechanism by which Gβ acts upstream of Gαi/Pins is not yet clear. Some studies have shown that LGN interacts with GDP-bound Gαi, and the interaction stability is regulated by resistance to inhibitor of cholinesterase 8 (Ric-8) (Figure 1C), a chaperon and non-canonical GEF for the Gα subunits (David et al., 2005). Accordingly, during asymmetric cell division, in order to form the Gα/GPR-1/2 complex, Ric-8, by its GEF activity, is needed to allow Gα subunit go through one round of GTP hydrolysis, therefore, Ric-8 mutants display several defects leading to a loss in the asymmetry of the spindle mitotic positioning (Hampoelz et al., 2005). Interestingly, Ric-8 has a crucial role during this process as its absence also disrupts the localization of Gα, Lin-5, GPR-1/2, and dynein (Woodard et al., 2010), suggesting a probably scaffold protein function (Toro-Tapia et al., 2018; Gabay et al., 2011; Klattenhoff et al., 2003). Correspondingly, in *C. elegans*, Ric-8 is essential to localize GPA-16 at the cell membrane by directly interacting with it (Afshar et al., 2005). As well as, in *Drosophila*, Ric-8, in addition to its GEF function on Gαi, is also required for Gαi plasma membrane localization, probably acting as a scaffold protein (Wang et al., 2005; Hampoelz et al., 2005; David et al., 2005).

On another hand, we found several examples supporting the essential role for Ric-8 and Gαi controlling cell division. Gαi isoforms (Gαi1-3) and regulator of G protein signaling 14 (RGS14) (a GAP for Gαi proteins) have been described to be localized at the centrosome in non-polarized HeLa cells (Figure 2D) (Cho and Kehrl, 2007), as well as, RGS14-Gαi-GDP-Ric-8A complex in mouse brain (Cho and Kehrl, 2007; Vellano et al., 2011). Another GEF for Gαi, Girdin (GIV) (Figure 1C), has been also found regulating other polarity processes. Specifically, GIV and Gαi3 are regulated by Par-3, which interacts with GIV, inducing tight junction formation and apical domain development, thus, promoting apicobasal polarity (Sasaki et al., 2015).

Together all these results rise the question, which is the specific role that Gαi plays during asymmetric cell division. For one side, as was mention above Gαi by its myristylation is localized at the plasma membrane (Chen and Manning, 2001; Morales et al., 1998), thus localizing the Gαi, LGN, dynein/dynactin complex and NuMA at the membrane, where together with Ric-8A, this latter acting as a GEF and scaffold protein, allowing the cycle between GDP-GTP that later by RGS and Gαi intrinsic GTPase activity hydrolyzes GTP to GDP, prompting the interaction between astral microtubules and cell cortex (di Pietro et al., 2016) (Figure 2A). Indeed, the association of Gαi with the spindle microtubules suggests that the G-protein subunit may regulate the assembly and disassembly of mitotic spindles by controlling microtubule assembly/dynamics. This insight, although not yet fully understood, provides a rational basis to understand the mechanism by which Gαi contributes to other biological processes through the control of microtubule dynamics and polarity establishment.

During development, symmetric cell division allows the cells to be cloned, whilst during asymmetric cell division different cells are originated in other to accomplish different functions. The last process, also contributes during adult life, specifically in physiological events like wound healing and tissue regeneration, cell differentiation, immune response and diseases, such cancer (Mascré et al., 2012; Aragona et al., 2017). Therefore, Gαi family and fate determinants are crucial to induce asymmetric cell divisions in order to create multiple type of cells contributing to tissue and organism diversity.

## Gαi regulates growth factor signaling pathways

**FIGURE 3:**
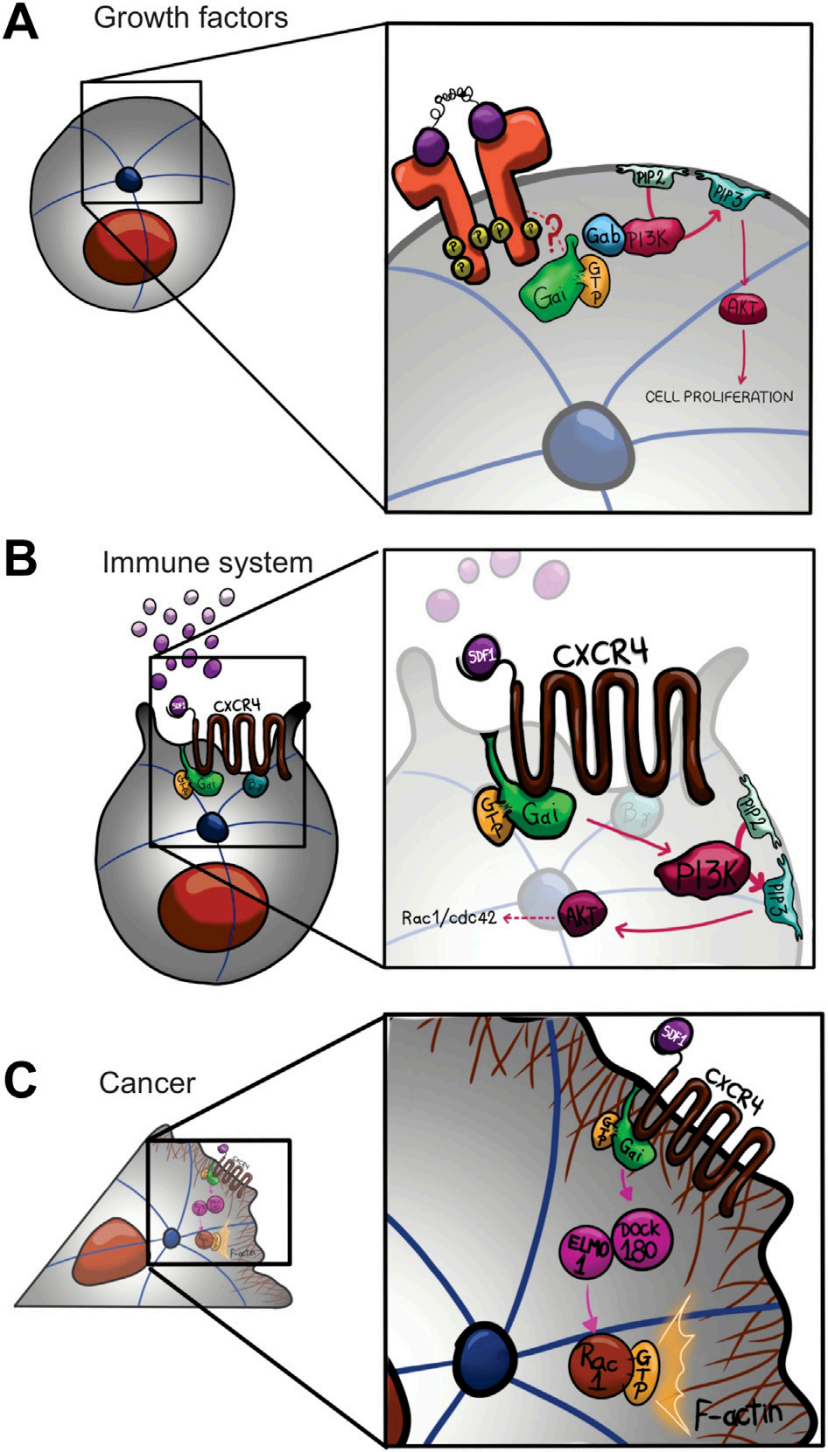
Gαi non-canonical signaling network models that show regulation of cell proliferation **(A)**, cell migration in immune system **(B)** and cancer **(C)**. There are multiple integrated pathways and crosstalk between Gi and tyrosine kinases signaling pathways regulating cell proliferation, growth, migration, and survival. All processes induced upon activation of receptor tyrosine kinases (RTKs), including epidermal growth factor receptor (EGFR), fibroblast growth factor receptor (FGFR), vascular endothelial growth factor receptor (VEGFR), and keratinocyte growth factor receptor (KGFR), activating PI3K-AKT pathway. RTK upon activation by its ligand is coupled to Gαi by an unknown mechanism, promoting the activation of PI3K-AKT signaling by Gαi/Gab interaction **(A)**. On another hand, CXCR4 (C-X-C chemokine receptor type 4) activated by SDF-1 is coupled to Gαi, promoting the activation of PI3K-AKT signaling accumulating D3-phosphoinositol lipids **(B)**. Finally, during invasive migration in breast cancer cells, Gαi2 in response to the same ligand, SDF-1, regulates the activation of Rac proteins through Elmo1/Dock180 interaction, contributing on the actin polymerization and migration **(C)**.

As mentioned above, Gαi protein subunits were originally characterized by their ability to inhibit adenylyl cyclase activity, and epinephrine, acetylcholine, dopamine, and serotonin have been used to stimulate physiological responses through Gαi protein subunits (Kindt et al., 2002; Heubach et al., 2004; Watts et al., 1998; Gou et al., 2010). Upon GPCR-ligand binding, Gαi proteins are activated and released from Gβγ subunits, which can now indirectly interact with PI3K, leading to the activation of several downstream effectors (Bondeva et al., 1998; Schwindinger and Robishaw, 2001; Zhang et al., 2015; Nůsková et al., 2021) (Figures 3A,B). Several studies have described Gβγ-PI3K signaling, specifically in the chemotaxis context, (Haastert, and Devreotes, 2004), although, few described Gαi association with PI3K, as we will describe bellow (Zhang et al., 2015; Nůsková et al., 2021). PTX ADP-ribosylates Gαi protein subunits at their C-terminus preventing their interaction with GPCRs and has been used as a molecular tool to determine the multiple cellular processes where Gαi proteins are involved, acting as signal transducer for GPCRs at the plasma membrane (Marrari et al., 2007). However, Gαi proteins are also found intracellularly, suggesting the possibility that these proteins perform cytoplasmic functions (Preininger et al., 2003; Marrari et al., 2007, Koelle, 2006). It has been described in mammals, the Src family tyrosine kinase members, acting as targets of Gαi proteins upon activation of GPCR, RTK, and non-RTK proteins (Ma et al., 2000), suggesting a crosstalk between Gi proteins and tyrosine kinase signaling pathways (Natarajan and Berk 2006). Accordingly, *in vitro* and *in vivo* assay demonstrated that c-Src tyrosine kinase interacts and is activated by the active conformation state of both Gαi and Gαs (Ma et al., 2000). Likewise, c-Src phosphorylates several Gα proteins included Gαi and Gαs on tyrosine residues enhancing G-protein function (Hausdorff et al., 1992). On another hand, Gαi proteins control cell proliferation, growth, migration, and survival by interacting with downstream effectors from receptor tyrosine kinases (RTKs), such as epidermal growth factor receptor (EGFR), vascular endothelial growth factor receptor (VEGFR), fibroblast growth factor receptor (FGFR), and keratinocyte growth factor receptor (KGFR), activating the phosphatidylinositol 3-kinase- protein kinase B-mammalian target of rapamycin complex 1 (PI3K-AKT-mTORC1) pathway (Figure 3A) (Cao et al., 2009; Liu et al., 2018; Zhang et al., 2015; Wang et al., 2014; Sun et al., 2018; Marshall et al., 2018). Indeed, Cao et al. (2009) showed that in response to epidermal growth factor (EGF), loss of Gαi1 and Gαi3 proteins inhibited cell proliferation and survival by decreasing the levels of cyclin D and by its mean decreasing the phosphorylation of AKT and mTORC1, thus impairing the interaction with downstream targets, such as glycogen synthase kinase 3 (GSK-3), forkhead box O (FoxO) transcription factor, eukaryotic translation initiation factor 4E (elF4E)-binding protein (4E4E-BP1), and ribosomal protein S6 (S6).

In the canonical heterotrimeric G-protein signaling pathway, Gi heterotrimers are activated by the GPCR, Gαi and Gβγ subunits are released, acting synergistically or in opposition toward tyrosine kinase signaling yields. On another hand, non-receptor GEFs, including GIV (Girdin), for Gαi have been discovered (Garcia-Marcos et al., 2009) (Figure 1C). In the cell migration during wound healing, macrophage chemotaxis, and tumor cell metastasis, all cellular processes triggered by growth factors (EGF and insulin), required the activation of Gαi by GIV, showing the need of Gαi-GTP state in order to activate downstream processes (Ghosh et al., 2008). In addition, GIV overexpression promotes colon and breast cancer metastasis (Jiang et al., 2008; Ling et al., 2011; Garcia-Marcos et al., 2011). Indeed, GIV directly interacts with both Gαi and EGFR, increasing EGFR autophosphorylation and extending its association to the membrane, thus activating cell migration by PI3-kinase-AKT and PLCγ1 signaling pathways (Garcia-Marcos et al., 2011). Accordingly, in GEF-deficient-GIV mutant cells, Gαi–GIV–EGFR complex cannot be assembled, EGFR autophosphorylation is reduced, and mitogenic signals, such as ERK1/2 and Src, are amplified, triggering cell proliferation (Ghosh et al., 2010). Together these evidences, show the crucial role for Gαi-GTP state, indeed Gβγ free, triggered either by receptor-GEF activation or GIV-activation in order to trigger these processes.

As we have been discussing here, Gαi plays a crucial role in growth factor signaling. Indeed, brain-derived neurotrophic factor (BDNF)-TrkB (tropomyosin-related kinase) receptor signaling, that mediate activity-dependent dendrite formation, via PI3K and mitogen-activated protein kinase (MAPK) (Dijkhuizen and Ghosh, 2005), is also regulated by Gαi proteins (McAllister et al., 1996; O’Neill et al., 2017; Marshall et al., 2018). In hippocampal neurons, Gαi1 and Gαi3 knockdown significantly reduces BDNF signaling and disrupts dendrite morphology, producing larger depressive behavior effects, demonstrating that both G-proteins are essential for TrkB receptor signaling and brain function (Marshall et al., 2018).

Finally, Gαi proteins have been also involved in the activation of PI3K-AKT-mTORC1 signaling by Gab1 (growth-factor receptor binding 2 [Grb2]-associated binding protein 1) in response to EGF (Cao et al., 2009; Liu et al., 2018; Zhang et al., 2015; Li et al., 2016; Nůsková et al., 2021) (Figure 3A). Therefore, Gαi proteins can control cellular growth, proliferation, and migration by modulating signaling through RTKs. For instance, cell proliferation and migration induced by keratinocyte growth factor- (KGF) and EGF are impaired upon Gαi1 and Gαi3 knockdown (Cao et al., 2009; Zhang et al., 2015). Accordingly, cells that are proliferating, such as wounded human skin or cancer cells, display an increase in the expression of Gαi1/3 [Zhang et al., 2015; Liu et al., 2018; Nůsková et al., 2021]. In summary, the Gαi proteins are critical to activate oncogenic signaling downstream of growth factor receptors that control cell proliferation and migration, although the detailed mechanism is yet to be fully elucidated.

## Gαi controls cell migration

Cell migration is a fundamental process involved in multicellular organisms to establish and maintain the proper organization of cells and tissues under variable physiological conditions. In adults, cell migration is essential to perform a proper tissue homeostasis, immune response, and wound repair, while changes in cellular motility are involved in the etiology of severe pathologies including cancer, atherosclerosis, defective immune response, and birth defects. Cell migration during tissue morphogenesis, requires changes in cell polarity by modifications at the cytoskeleton organization, upon mechanical and chemical cues from the surrounding environment (Schwarz and Gardel, 2012; Abercrombie et al., 1971; Roubinet et al., 2012). For instance, during an immune response, the chemical signals generated by the interaction between chemoattractants with their receptors direct the localization of leukocytes toward several tissues and peripheral organs to mediate inflammation. In this context, most chemoattractants and chemokines signal through GPCRs that couple with Gi, dissociating Gαi subunit from its associated Gβγ heterodimers (Hepler and Gilman, 1992; Neer, 1995) to activate downstream effectors in order to control immune cell migration (Arai et al., 1997; Neptune et al., 1997; Neptune et al., 1999). Gαi2 and Gαi3 are highly expressed in the immune system (Han et al., 2005), thus, Gαi signaling is involved during many leukocyte biological functions, among these we found, macrophage phagocytosis and migration (Lee et al., 2009; Weiss-Haljiti et al., 2004), T and B cell migration towards the lymph nodes (Han et al., 2005; Hwang et al., 2007), eosinophils migration to sites of allergic tissue injury (Pero et al., 2007), and neutrophils migration during acute inflammation (Pero et al., 2007; Zarbock et al., 2007).

Several molecular tools together with *in vivo* analysis have allowed to understand Gαi functions during immune cells migration. For example, using knockout animals, cell knockdown and rescue experiments, together with microscopy tools revealed the essential role of Gαi2 during homeostatic and inflammation-induced migration by controlling actin cytoskeleton remodeling and chemotactic migration of macrophages (Wiege et al., 2012). Gαi2^−/−^ mice displays defects in the signaling upon B cell chemokine receptor, causing depressed B cell chemotaxis and poor B cell adherence to lymph node high endothelial venules (HEVs), defects that were not rescued by Gαi1 and Gαi3 (Han et al., 2005). The use of knockdown and knockout cellular models has also revealed that different Gαi subtypes may play distinct roles. For example, Gαi2 and Gαi3 expressed at T lymphocytes act differently to regulate cell migration. Indeed, the lack of Gαi2 function in T cells, impairs migration mediated upon stimulation of C-X-C chemokine receptor type 3 (CXCR3) by chemokine C-X-C motif ligand 9, 10, 11 (CXCL9, CXCL10, or CXCL11) (Thompson et al., 2007). For the contrary, the lack of Gαi3 function in T cells increases migration upon CXCR3 stimulation, suggesting that this Gαi isoform may be a negative regulator of migration (Thompson et al., 2007).

It is well-established that C-X-C chemokine receptor type 4 (CXCR4) is coupled to Gαi, promoting the activation of PI3K (Figure 3B) (Dutt et al., 1998; Curnock et al., 2003). Specifically, stromal derived factor 1 (SDF) activate migration by a molecular mechanism mediated by Gαi-proteins, the tyrosine kinases, Src and IL2-inducible T cell kinase (ITK), as well as PI3K (Figure 2B) (Fischer et al., 2004). In Jurkat T cells upon SDF-1-CXCR4 stimulation, Gβγ is released from Gαi, triggering molecular downstream effector to promote cell migration (Tan et al., 2006). Likewise, it has been described a similar mechanism for lysophosphatidic acid receptor 2 (LPA2 receptors). Specifically, LPA (lysophophatidic acid)-induced migration of CAOV-3 ovarian cancer cells involved activation of the Gαi/SRC/EGFR/ERK signaling axis (Jeong et al., 2008). In the same context, Ward and Dhanasekaran, 2012 demonstrated a critical role for Gαi2 in LPA-stimulated cell migration, by regulating the tyrosine phosphorylation of the scaffold protein, p130 Crk associated substrate (p130Cas), to induce metastasis in ovarian cancer cells. In breast cancer cells, Gαi2 regulates the activation of Rac proteins through the GEF activity of Elmo1/Dock180 (Engulfment and cell motility 1/dedicator of cytokinesis) interaction, contributing on the actin polymerization and migration (Figure 3C) (Li et al., 2013).

Activated Gαi also directly impacts neutrophil migration (Surve et al., 2016) by regulating adhesion through a cAMP-independent mechanism. Gβγ activation using the small molecule 12,155 inhibited cell migration, altered the cell polarity, and increased the adhesion of neutrophils (Surve et al., 2016). In the same context, a Gαi1 constitutively active mutant, Gαi1 (Q204L), rescued the loss of migration phenotypes caused by Gβγ activation (Surve et al., 2016). Specifically, under Rap1a-Radil (Ras related protein 1a-Ras associating and dilute domain-containing protein) signaling pathway, Gαi1Q204L but not the wild type Gαi1 is sufficient to rescue the neutrophil morphology, from an elongated phenotype to circularly cell shape, suggesting that active Gαi can regulate cell rear retraction, critical process during cell migration (To and Smrcka, 2018). Therefore, Gαi has critical and varied functions regulating cell migration depending on the physiological context, through releasing Gβγ, tyrosine kinase regulation and also by regulating small G protein family, such Rac, in order to control cytoskeleton organization, although the detailed mechanism still an exciting topic to understand.

## Gαi function during development

By 1993, the first *in vivo* knockdown of Gαi2 in mice revealed further insights into the possible roles of G proteins in early mammalian development (Moxham et al., 1993). Indeed, in totipotent mouse F9 teratocarcinoma cells, Gαi2 reduction by retinoic acid promotes formation of a primitive endoderm, whereas Gαi2 activation blocks the formation of a primitive endoderm (Watkins et al., 1992). Also, wingless-related integration site (Wnt)-mediated signaling through the GPCR, Frizzled, is PTX-sensitive in mouse F9 teratocarcinoma cells (Liu et al., 2001), *zebrafish* and *Xenopus* embryos (Slusarski et al., 1997), and *Drosophila melanogaster* (Katanaev et al., 2005), suggesting that Gαi has crucial roles during early development. In 2016, our group demonstrated that Gαi2 transcript is expressed at early neurula stages in *Xenopus* embryos within neural and neural crest tissue. Specifically, at embryo stages 23 and 24, Gαi2 transcript is displayed at the presomitic mesoderm and at the front of the embryos, region which later differentiate into the brain and neural tube. From stage 27, Gαi2 transcript is expressed in neural crest routes and placodes, and in vascular tissue derivatives including the posterior cardinal and intersomitic (Fuentealba et al., 2016). Interestingly, Gαi2 transcript was also expressed at the dorsal marginal zone, a region critical to neural crest induction (Fuentealba et al., 2016).

In mice, loss of Gαi2 function inhibits the insulin receptor tyrosine kinase signaling by reducing the levels of phosphotyrosine phosphatase (Moxham and Malbon, 1996). Also, in mice, retinal pigment epithelium formation is controlled by Gαi family proteins, specifically Gαi3 (Young et al., 2013). In addition, Gαi influences osteoblast differentiation, which is induced by CXCR4 (Zhu et al., 2007), as well as osteoblast proliferation and survival induced by LPA (Grey et al., 2001). Interestingly, Gαi3 is also involved during normal patterning of the axial skeleton, thus its expression is critical in sclerotomal derivatives and Gαi1 and Gαi2 are able to rescue partially the loss of Gαi3, although depending on genetic background (Plummer et al., 2012).

As we mentioned above, three isoforms from the Gαi family have been described, including Gαi1, Gαi2 and Gαi3. However, Gαo another member of the PTX-sensitive Gαi/o family, have been reported to be involved in the central nervous system (CNS) development. Gαo is the most abundant isoform in the CNS, although, its role remains to be elucidated. Nevertheless, recent studies have found that global deletion of Gαo impairs cerebellar cortical development in mice (Cha et al., 2019). Specifically, depletion of Gαo induced cerebellar hypoplasia and reduced arborization and dendritic spines of Purkinje cell dendrites from the inferior olivary nucleus in mice (Cha et al., 2019).

In *C. elegans*, Gαo has been involved during neuronal migration in early development. Indeed, the neurotransmitter, serotonin, induce neuronal migration by stimulation of GPCR/Gαo signaling (Kindt et al., 2002). Gαo also regulates the migration of neurons in moth (Horgan et al., 1994), as well as during growth cones of developing neurites in cultured human pheochromocytoma PC12 cells (Strittmatter et al., 1990). In *Drosophila*, Gαo is asymmetrically localized in cells, and together with Pins, regulates Frizzled-mediated asymmetric cell division and planar cell polarity (Katanaev et al., 2005; Katanaev and Tomlinson, 2006). Although, these findings support the crucial and pleiotropic function that Gαi signaling pathways have during development, the detailed mechanism(s) and role of other signaling cascades remain to be elucidated.

## Conclusion and future perspectives

In this review, we have briefly summarized the activation, signaling, and physiological functions of the Gαi subunit from heterotrimeric G proteins. Gαi proteins are well known to transduce signals between GPCRs and their downstream effectors in response to extracellular ligands (Birnbaumer, 2007). Nevertheless, several studies indicate that Gi protein function during establishment of cell polarity and asymmetric cell division may not involve any extracellular signal (di Pietro et al., 2016). Although, Gαi acts with several other proteins (e.g., AGS and GEFs) to regulate these processes, many questions remain to be answered regarding the detailed mechanism of Gαi-protein regulation of astral microtubules pulling forces during cell division. Several evidences showed that tubulin could be a direct downstream target of G-proteins in the context of cell division (Willard et al., 2004), and possible in other biological processes such as cell migration and wound healing. This is supported by evidence that both Gα and Gβγ subunits can bind directly and regulate microtubule dynamics *in vivo* and *in vitro* (Chen et al., 2003; Popova and Rasenick, 2003; Roychowdhury et al., 1999; Roychowdhury and Rasenick, 1997; Wang et al., 1990; Sarma et al., 2003). Indeed, activated state GTP-bound G*α*i1 can directly interact with tubulin, transactivating its intrinsic GTPase activity and modulating microtubule dynamics (Chen et al., 2003; Roychowdhury et al., 1999). In addition, Lin-5 and GPR-1 interact with dynein, a motor protein that moves along microtubules transporting various cellular cargos and providing forces during mitosis, to control spindle positioning, suggesting that Gαi could regulate the spindle pulling forces through interaction with Lin-5/GPR-1 and dynein (Couwenbergs et al., 2007).

In cancer, embryonic development, and cell growth, tyrosine kinases play an important role in cell division and proliferation. As was mentioned here, c-Src, a non-receptor tyrosine kinase phosphorylates specific tyrosine residues in other downstream tyrosine kinases to regulate these processes. Several studies described here also indicate possible direct interactions between Gα subunits and tyrosine kinases (Luttrell and Luttrell, 2004), supporting the idea that Gαi is able to activate tyrosine kinases signaling in a direct manner. Furthermore, c-Src is also involved in cell adhesion during migration. Inhibition of c-Src induces loss of cell adhesion and membrane blebbing, affecting cell migration (Logue et al., 2018). There is also substantial evidence supporting that Src family kinases can regulate the activity of Gα subunits through tyrosine phosphorylation as it phosphorylates Gαs, Gαi1, Gαi2, and Go *in vitro*, suggesting that Gα subunits are potentially involved in cell adhesion and migration (Hausdorff et al., 1992). As we mentioned above, it has been described a novel role for Gαi1-GTP regulating cell adhesion during neutrophils migration in a Rap1a-Radil manner, which likely functions through c-Src activity (To and Smrcka, 2018).

Although the Gαi family was first classified as inhibitors of adenylyl cyclase, new data have reveled non-canonical functions that impact cell behavior. However, several interesting questions remain to be answered. For example, could Gαi control cell polarity during cell migration in development using the same mechanism used in cell division (i.e., controlling the interaction between microtubules to induces morphology changes). Given that Gαi subunits are highly similar (e.g., Gαi1 and Gαi3) with around 94% sequence identity, they clearly perform different functions, demonstrating the complex mechanisms underlying Gαi signaling pathways. Thus, further studies are required to systematically dissect the multiple signaling mechanisms that regulate cell behavior.
